# Gouty tophus as a rare cause of a Hepple stage V osteochondral lesion of the talus

**DOI:** 10.1186/s40001-021-00597-5

**Published:** 2021-10-19

**Authors:** Bo Tang, Cheng Fan

**Affiliations:** Sports Medicine Center, First People’s Hospital of Xining City, Xining, 810000 Qinghai China

**Keywords:** Gout, Talus osteochondral lesion, Diet, Young patient, Treatment

## Abstract

**Background:**

Talus osteochondral lesion is commonly associated with trauma, avascular necrosis or even genetic factors, but gouty tophus as a cause of Hepple stage V type talus osteochondral lesion is rare.

**Case presentation:**

Here, we report a case of an 18-year-old man who complained of left medial deep ankle pain on ambulation. This young man had an extreme liking of sea food rich in purines and also sugar-sweetened drinks. He was diagnosed with a Hepple stage V type talus osteochondral lesion and was treated with medial malleolus osteotomy and an osteochondral graft. The talus osteochondral lesion was found to be a gouty tophus and was completely removed. Hypouricemic therapy was prescribed for 2 months, which allowed the patient to walk with a visual analogue score (VAS) score of 1. He was followed up for 12 months.

**Conclusions:**

Young people with an extreme liking of sea food rich in purines and also sugar-sweetened drinks may be at a risk of developing gout. Acute onset of ankle atraumatic pain, swelling with a high level of serum uric acid and a talus osteochondral lesion with cyst formation should make physicians consider a diagnosis of gout.

## Background

Talus osteochondral lesion is characterized by damage to the articular cartilage of the talus and its underlying subchondral bone and can result in persistent ankle pain or osteoarthritis [1]. If conservative therapy such as non-steroidal anti-inflammatory drugs, hyaluronic acid or platelet-rich plasma injection, or limited weight-bearing failed, surgical intervention mainly including arthroscopic debridement, microfracture, osteochondral autologous or allograft transplantation is the treatment of choice [2]. The Hepple classification has been widely used for the diagnosis and treatment of talus osteochondral lesion. Combination therapy of arthroscopic debridement or microfracture, and hyaluronic acid or platelet-rich plasma injection are mainly used for I to IV type lesions, and osteochondral grafts are often required to manage satisfactorily a Hepple stage V type lesion, which refers to subchondral cyst formation [3]. Talus osteochondral lesions have been reported to be commonly correlated with foot and ankle trauma, avascular necrosis or genetic factors [2], but gouty tophus as a cause of a Hepple stage V type talus osteochondral lesion is rare. Historically, gout was commonly seen in the aged population, with few young people suffering from this condition. Here, we report an 18-year-old male patient with a severe Hepple stage V type talus osteochondral lesion caused by a gout tophus that was successfully treated with an autologous graft and hypouricemic therapy combined with diet modification.

## Case presentation

**Fig. 1:**
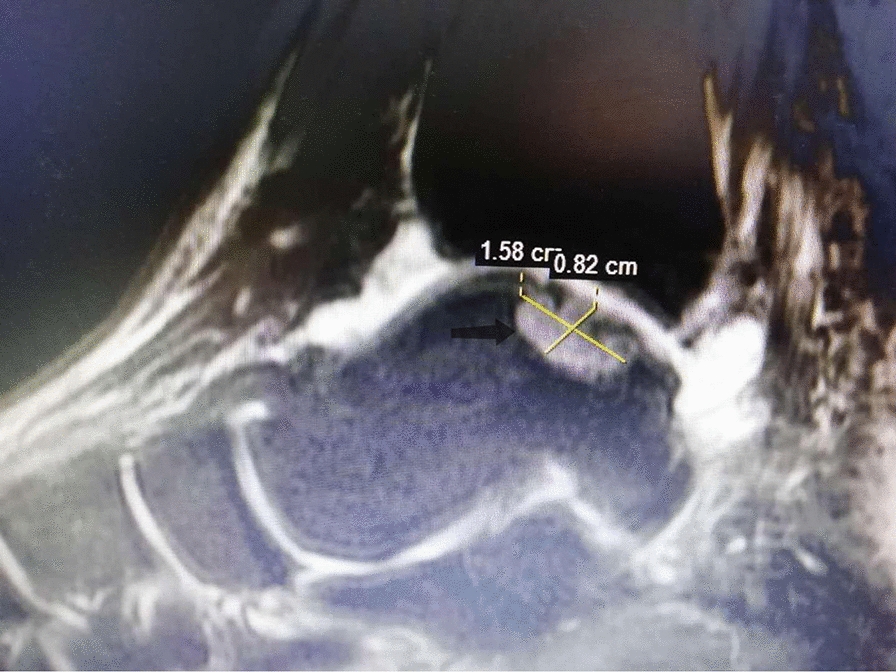
Ankle MRI of the 18-year-old young male patient. T2-weighted sagittal view of the ankle MRI revealed a 0.94 × 0.82 × 1.58 cm, well-defined, cystic structure in medial, central talus (arrow)

**Fig. 2:**
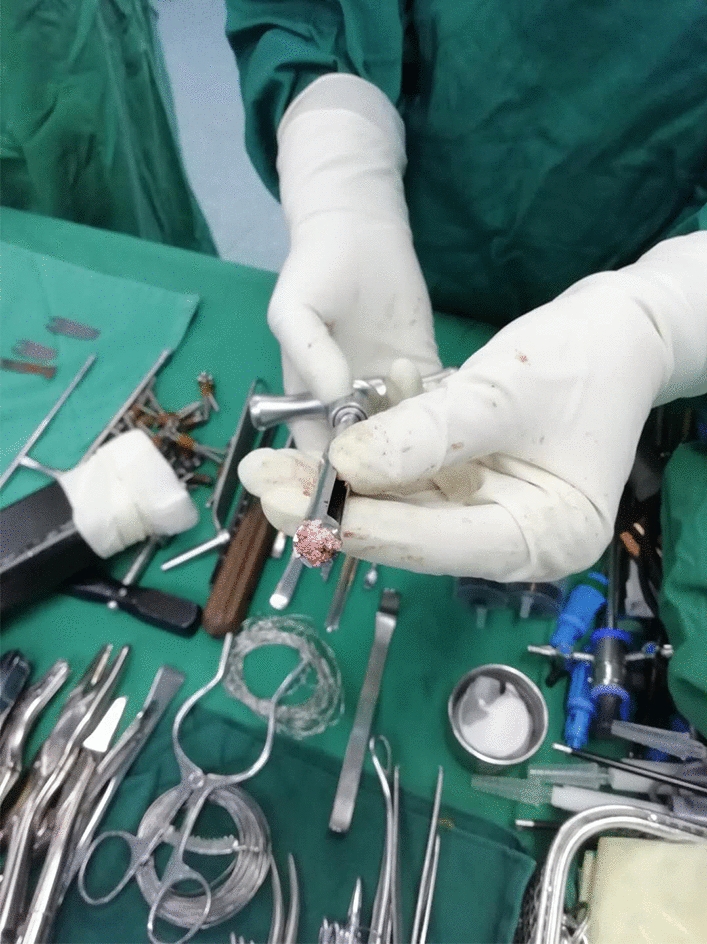
The talus osteochondral lesion was removed with trephine. White gouty tophus removed with a trephine was found during the operation

**Fig. 3:**
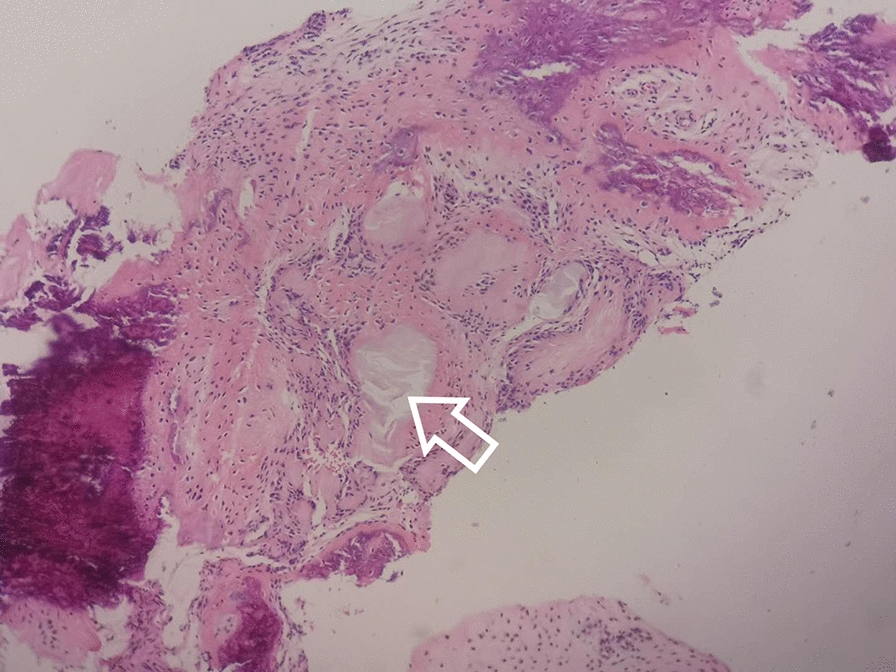
Postoperative histological results of the removed lesions. Monosodium urate monohydrate (MSU) crystals (white arrow) were found in postoperative hematoxylin and eosin-stained histological sections

A 172-cm tall, 18-year-old man, weight 100 kg, was admitted to our department in January 2020 due to left medial deep ankle pain after stamping the clutch about 10 days ago, who had an extreme liking of seafood, beer and carbonated drinks. During the physical examination, medial ankle tenderness and mild swelling were present, with a VAS score of 6 or 7, but the range of ankle motion was normal. Ankle X-rays, computerized tomography (CT) and magnetic resonance imaging (MRI) scans were conducted and a medial talus osteochondral lesion with subchondral cyst formation was detected (Fig. 1). Various laboratory tests detected high levels of uric acid (568 µmol/L), anti-streptolysin O (130.10 IU/mL), procalcitonin (0.28 ng/mL) and a high level of serum C-reactive protein (39.50 mg/L). The patient’s erythrocyte sedimentation rate, blood cell counts, and other biochemical indexes were all within the normal range. He was primarily diagnosed with a Hepple stage V type talus osteochondral lesion, but the etiology was not clear. An ankle arthroscopy was conducted, and we found a few gouty tophus deposits in the talus articular cartilage and synovial tissue. The medial, central talus articular cartilage was soft, uneven and partially defective, with gouty tophus deposits. A 1.5-mm K-wire was drilled into this site to confirm the talus lesion site under C-arm fluoroscopy. A medial curved incision was made on the medial malleolus and the talus osteochondral lesion was exposed after inverted V-shaped medial malleolus osteotomy and removed with trephine; the removed lesions were found to be gouty tophus (Fig. 2), which was further confirmed by the finding of monosodium urate monohydrate (MSU) crystals in postoperative histological sections (Fig. 3). After complete debridement of these lesions, the lesion site was not a regular shape, and a very small amount of normal osteochondral tissue was cut with a small osteotome to form a square lesion site. Then an osteochondral block of the same size was harvested from the autologous talus neck and grafted onto the site and fixed with two 2.0 mm bioabsorbable screws. The donor site was filled and tamped with the cancellous bone on the osteotomy surface of the medial malleolus. Then the medial malleolus was fixed with three cannulated screws after reduction. Aceclofenac dispersible tablets and allopurinol were taken by the patient immediately after the operation and he was advised to use his left ankle passively, with no weight-bearing for 5 weeks. At this time his serum uric acid concentration was in the normal range and a good medial malleolus union was revealed by X-ray imaging. Partial weight-bearing was encouraged 2 months postoperatively when the cyst talus osteochondral lesion had disappeared and was replaced with normal bone tissue 6 months postoperatively. The patient could walk with slight pain at this time with a VAS score of 1 and was very satisfied with the outcome. Regular monitoring of serum uric acid concentrations and his modified diet continued, and he has been followed up for 12 months.

## Discussion

The Hepple stage V type talus osteochondral lesion was mainly managed using an osteochondral grafting technique, with positive results having been reported in several previous studies [4, 5]. It is noteworthy, however, that this case of a Hepple stage V type talus osteochondral lesion was caused by gout tophus. An osteochondral graft harvested from the autologous talus neck combined with hypouricemic therapy and diet modification produced a satisfactory clinical outcome.

It has been reported that gout is commonly diagnosed in patients aged > 60 years of age [6]. The present case was an 18-year-old male with a severe talus osteochondral lesion caused by gout tophus, which may have developed due to his bad dietary habit; he told us that he had an extreme liking for seafood, beer and carbonated drinks, all known risk factors for gout [7, 8]. In the literature, we found a similar report on gout tophus as an unusual cause of talus fracture, but this was a 41-year-old man with a 20-year history of untreated gout, and pathological talus fracture-like neoplastic bone destruction was also reported [9], whereas in the present case the cyst lesion was well defined. We also found a remarkably similar report on gout tophus as an unusual cause of talus osteochondral lesion in a 16-year-old male who complained of fluctuating ankle pain on the anteromedial side, which was successfully managed with arthroscopic debridement and microfracture [10]. In the present case, osteochondral autologous transplantation was employed mainly due to the relatively larger lesion size. A systematic review has suggested that microfracture may best be reserved for lesion sizes less than 107.4 mm^2^ in area and/or 10.2 mm in diameter [11]. However, a recent review also indicated that no consensus has been reached regarding the treatment of osteochondral lesions of the talus due to limited high-level evidence [12].

In addition, the question remains why this patient did not suffer a gout flare previously when the severe talus osteochondral lesion had occurred, and why the gout tophus was concentrated on the talus subchondral region. It should be mentioned that during his operation gout tophus was found to be deposited on the ankle synovial and talus articular cartilage. Further research will be required to clarify the underlying etiology of a severe, localized talus osteochondral lesion caused by gout tophus. Nevertheless, we present this case to raise attention to the previous diet of this young man which was rich in purines, and also his excessive consumption of beer and sugar-sweetened carbonated drinks. This was the probable reason for the development of the severe talus osteochondral lesion in this patient at such a young age.

## Conclusions

Although gout is mainly diagnosed in the aged population, young people who like extreme diets that include sea food rich in purines, beer and sugar-sweetened carbonated drinks may be at risk of prematurely developing gout.

A severe talus osteochondral lesion occurred in our young patient without previous gout flares. An acute onset of ankle atraumatic pain, swelling with a high concentration of serum uric acid and a talus osteochondral lesion with cyst formation should make a physician think of gout as the diagnosis.

A Hepple stage V type talus osteochondral lesion due to gouty tophus can be successfully managed with an autologous osteochondral graft combined with uric acid-lowering therapy and diet modification.

## Data Availability

Data sharing not applicable to this article as no datasets were generated or analyzed during the current study.

## Abbreviations

VAS: Visual analogue score
MSU: Monosodium urate monohydrate
CT: Computerized tomography
MRI: Magnetic resonance imaging
